# The complete chloroplast genome sequence of *Michelia wilsonii*: an endangered species in China

**DOI:** 10.1080/23802359.2020.1721354

**Published:** 2020-02-03

**Authors:** Yongkang Sima, Yunqing Li, Xiaolong Yuan, Yi Wang

**Affiliations:** Laboratory of Forest Plant Cultivation and Utilization, Yunnan Academy of Forestry and The Key Laboratory of Rare and Endangered Forest Plants of State Forestry Administration, Kunming, Yunnan, People’s Republic of China

**Keywords:** *Michelia wilsonii*, chloroplast, Illumina sequencing, phylogenetic analysis

## Abstract

The first complete chloroplast genome (cpDNA) sequence of *Michelia wilsonii* was determined from Illumina HiSeq pair-end sequencing data in this study. The cpDNA is 160,100 bp in length, contains a large single-copy region (LSC) of 88,162 bp and a small single-copy region (SSC) of 18,786 bp, which were separated by a pair of inverted repeats (IR) regions of 26,576 bp. The genome contains 132 genes, including 87 protein-coding genes, 8 ribosomal RNA genes, and 37 transfer RNA genes. Further phylogenomic analysis showed that *M. wilsonii* closed to *Michelia odora* and *Michelia yunnanensis* in *Michelieae* tribe of the Magnoliaceae family.

*Michelia wilsonii* Finet et Gagnep [synonymous with *Michelia sinensis* Hemsl. et E. H. Wilson and *Magnolia ernestii* Fiylar] is the species of the genus *Michelia* belonging to the family Magnoliaceae (García 2012). *Michelia wilsonii* is one of the 120 species of small population of wild plants in China and it is an endangered species in China (Sun and Han 2019). The timber of *M. wilsonii* is a good material for landscaping, furniture, and construction (Xiao et al. 2019). The flowers and leaves of *M. wilsonii* contain essential oil, which had good fresh-keeping effect and bacteriostatic effect (Liu et al. 2008). Their bark and flowers can be used as drugs to inhibit bacteria, remove stasis and generate new, activate blood, and relieve pain (Zhu et al. 2015). However, there has been no genomic studies on *M. wilsonii*.

Herein, we reported and characterized the complete *M. wilsonii* plastid genome. The GenBank accession number is MN897729. One *M. wilsonii* individual was collected from Kunming arboretum, Yunnan Academy of Forestry, Yunnan Province of China (25°14′16″N, 102°75′21″E). The specimen is stored at Yunnan Academy of Forestry Herbarium, Kunming, China, with the accession number S99247. DNA was extracted from its fresh leaves using DNA Plantzol Reagent (Invitrogen, Carlsbad, CA, USA).

Paired-end reads were sequenced by using Illumina HiSeq system (Illumina, San Diego, CA). In total, about 9.7 million high-quality clean reads were generated with adaptors trimmed. Aligning, assembly, and annotation were conducted by CLC de novo assembler (CLC Bio, Aarhus, Denmark), BLAST, GeSeq (Tillich et al. 2017), and GENEIOUS v 11.0.5 (Biomatters Ltd, Auckland, New Zealand). To confirm the phylogenetic position of *M. wilsonii*, other 10 species of *Michelieae* tribe from NCBI were aligned using MAFFT v.7 (Katoh and Standley 2013). The auto algorithm in the MAFFT alignment software was used to align the 13 complete genome sequences and the G-INS-i algorithm was used to align the partial complex sequences. The maximum likelihood (ML) bootstrap analysis was conducted using RAxML (Stamatakis 2006); bootstrap probability values were calculated from 1000 replicates. *Liriodendron tulipifera* (MK477550) and *Liriodendron chinense* (KU170538) were served as the out-group.

The complete *M. wilsonii* plastid genome is a circular DNA molecule with the length of 160,100 bp, contains a large single-copy region (LSC) of 88,162 bp and a small single-copy region (SSC) of 18,786 bp, which were separated by a pair of inverted repeats (IR) regions of 26,576 bp. The overall GC content of the whole genome is 39.2%, and the corresponding values of the LSC, SSC, and IR regions are 37.9%, 34.3%, and 43.2%, respectively. The plastid genome contained 132 genes, including 87 protein-coding genes, 8 ribosomal RNA genes, and 37 transfer RNA genes. Phylogenetic analysis showed that *M. wilsonii* closed to *Michelia odora* and *Michelia yunnanensis* in *Michelieae* tribe (Figure 1). The determination of the complete plastid genome sequences provided new molecular data to illuminate the *Michelieae* tribe in Magnoliaceae family evolution.

**Figure 1.**
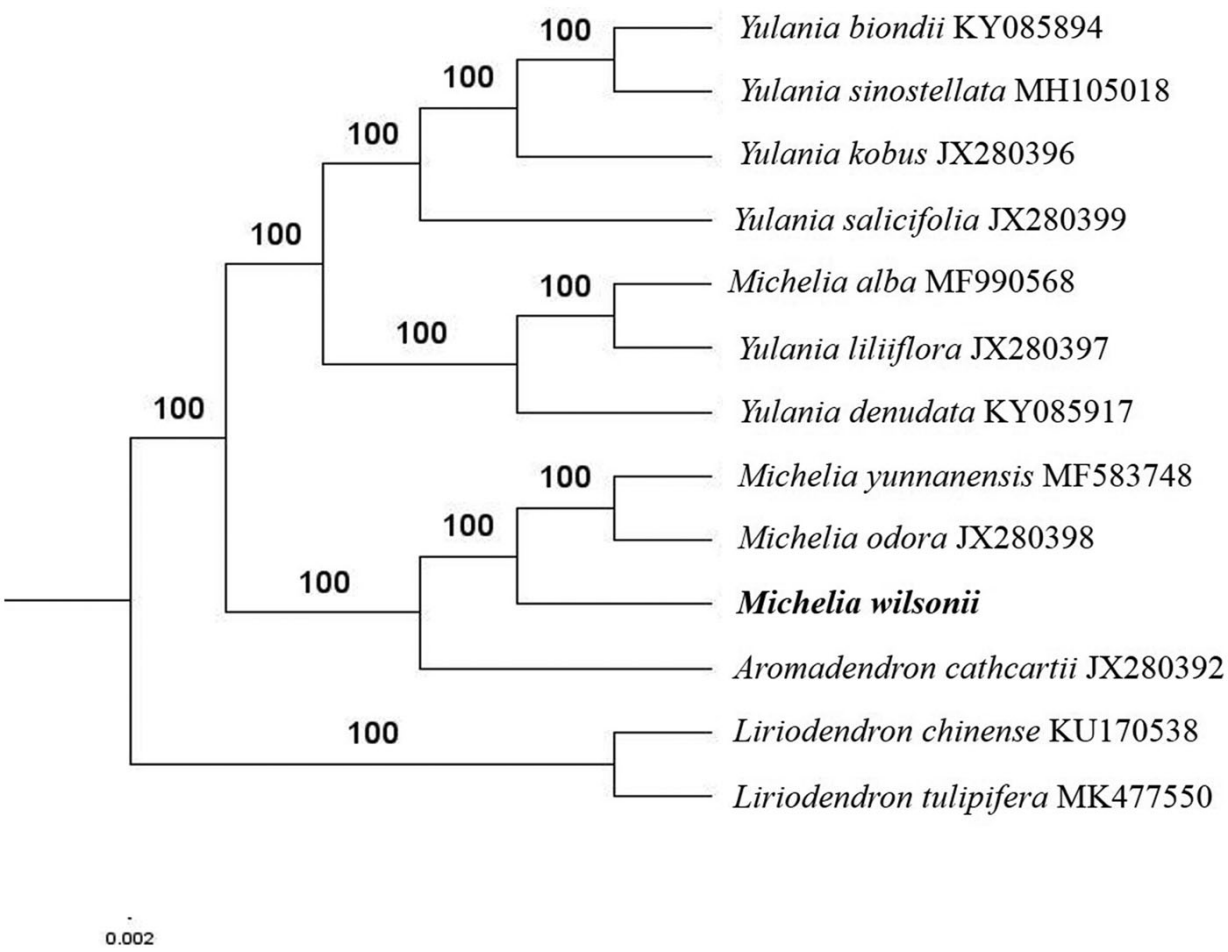
The maximum-likelihood tree based on the eleven chloroplast genomes of *Michelieae* tribe in Magnoliaceae family. The bootstrap value based on 1000 replicates is shown on each node.
